# Translational Entropy-Driven Competitive and Additive Effects on DNA Higher-Order Structure via Ion Exchange Between Cations of Different Valencies

**DOI:** 10.3390/e28060686

**Published:** 2026-06-13

**Authors:** Takahiro Kenmotsu, Haruto Ogawa, Takashi Nishio, Kenichi Yoshikawa

**Affiliations:** 1 Faculty of Life and Medical Sciences, Doshisha University, Kyoto 610-0394, Japan; cygn0005@mail4.doshisha.ac.jp; 2 Molecular Biosystems Research Institute, National Institute of Advanced Industrial Science and Technology (AIST), Ibaraki 305-8566, Japan; nishio-t@aist.go.jp; 3 Organization for Research Innovation, Doshisha University, Kyoto 610-0394, Japan; keyoshik@mail.doshisha.ac.jp

**Keywords:** translational entropy, ion exchange, counterions, multivalent cations, DNA conformational transitions, competitive and additive effects, counterion condensation

## Abstract

DNA conformational transitions in aqueous environments are strongly influenced by electrostatic interactions with surrounding cations. This review/perspective article summarizes the experimental findings reported during the last decade on the competitive/cooperative effects of cations with different valencies on DNA conformational behavior. Recent experimental studies based on single DNA observations have shown that divalent cations, such as Mg(2+) and Ca(2+), can inhibit DNA compaction induced by the trivalent cation spermidine (SPD(3+)), revealing that the effects of coexisting cations are not simply additive. Such competitive behavior cannot be adequately explained within the conventional Debye–Hückel framework, which predicts always additive electrostatic screening contributions from cations of different valencies. To elucidate the underlying mechanism of competitive effects, a theoretical framework has been proposed by extending the framework of current counterion condensation theory, which incorporates changes in translational entropy arising from the ion-exchange process between monovalent counterions and divalent or trivalent cations interacting with DNA as a highly negatively charged polyelectrolyte. In the theoretical framework, the increase in translational entropy arises from the ion exchange process between monovalent counterions and trivalent cations in the absence of divalent cations, whereas the presence of divalent cations diminishes the entropic gain associated with this exchange. By interpreting the recent experimental findings through the aid of the development of theoretical modeling, this review/perspective article provides a coherent insight on how coexisting multiple cations regulate DNA conformation.

## 1. Introduction

DNA is a highly negatively charged polyelectrolyte characterized by a persistence length of approximately 150 base pairs (bp), corresponding to about 50 nm [1,2], in aqueous and cellular environments. A long DNA chain on the order of several tens of kbp behaves as a semi-flexible chain [3,4] and adopts an elongated coil conformation under translational and intrachain Brownian motion in aqueous environments in vitro. Negatively charged DNA is electrostatically associated with a variety of cationic species in cells, including histone proteins, metal cations, and polyamines, which promote the compaction of long DNA chains [5,6,7,8,9,10,11,12,13,14]. Polyamines are ubiquitous polycationic molecules present in both prokaryotic and eukaryotic cells and play essential roles in a wide range of biological processes, including cell growth and proliferation [15,16,17,18]. A semi-flexible DNA chain adopts a variety of conformations in response to its surrounding environment. The conformational transition of DNA from an elongated coil to a compact state has been extensively studied both experimentally [11,12,19,20] and theoretically [21,22,23,24]. It has been confirmed that polyamines such as spermidine (SPD(3+)) and spermine (SPM(4+)) cause a large discrete phase transition for long individual DNA molecules, accompanied by a change in the effective segment density on the order of 10^4^–10^5^ [25,26]. Regarding the interaction between a highly charged polyelectrolyte and its counterions, Osawa developed a theoretical framework based on statistical thermodynamics [27]. Manning provided a phenomenological theoretical model for a linear polyelectrolyte known as counterion condensation theory [28]. The Manning–Oosawa counterion condensation theory suggests that the fraction of DNA negative charge neutralized by condensed counterions is expressed as θ=1−1/Zξ, where ξ is the Manning parameter (≈4.2 for double-stranded B-DNA) and *Z* is the valence of surrounding cations. The mechanism of the coil-globule transition of DNA molecules in good solvents has been argued intensively in relation to counterion condensation [3,29,30,31,32,33]. Building on these studies, Loh et al. proposed a theoretical framework for the free energy of a flexible polyelectrolyte chain that explicitly considers the translational entropy of counterions and evaluates their contribution to the conformational changes in the chain [34]. However, these theoretical frameworks do not fully account for the competitive effects observed in systems with multiple coexisting cations. The higher-order structural changes in DNA are expected to be closely related to the self-regulation of replication, transcription, and genetic activity in living cells [5,35,36,37,38]. In recent studies, the effects of higher-order DNA structural changes induced by biological polyamines, such as SPD(3+), on gene expression in cell-free systems have been reported using single-molecule fluorescence microscopy and atomic force microscopy (AFM) [39,40,41,42,43,44]. These studies have revealed that polyamine spermidine (SPD(3+)) exerts opposite effects, enhancement and inhibition, on gene expression depending on its concentration. This behavior has been discussed in the context of DNA conformational changes. Notably, at intermediate concentrations, a flower-like structure exhibiting a uniquely shrunken conformation with a parallel alignment of DNA strands was observed, and this structural state promoted gene expression. In contrast, gene expression is completely inhibited at higher concentrations of the polyamines, which are associated with tightly compacted DNA conformations.

## 2. Recent Studies Concerning the Effects of Cations on DNA Molecules

DNA is a highly charged polyelectrolyte, and its conformational stability, mechanical properties, and higher-order organization are critically governed by interactions with surrounding cations. Owing to the negative charge carried by each phosphate group in the DNA backbone, electrostatic repulsion along the chain is partially screened and neutralized by counterions in aqueous solution. The classical counterion condensation concept and subsequent thermodynamic treatments established that cations reduce the effective charge density of DNA and thereby modulate duplex stability, ligand binding, bending flexibility, and intermolecular interactions [45,46]. Experimental and computational studies have further shown that the influence of cations depends not only on their concentration but also on their valency, ionic radius, hydration state, and local binding preference [47,48].

Monovalent cations such as Na(+) and K(+) mainly stabilize DNA by screening the electrostatic repulsion between negatively charged phosphate groups. In this regime, the ion atmosphere surrounding DNA remains largely dynamic and diffuse, and monovalent salts generally promote duplex stability under ordinary bulk conditions [49,50]. Molecular dynamics simulations have shown that Na(+) and K(+) distribute preferentially around phosphate groups and grooves while retaining substantial mobility around B-DNA [51]. However, monovalent ions are not completely equivalent: Na(+) tends to condense more strongly around DNA than K(+) because of differences in electrostatic, steric, and hydration interactions [52]. Recent single-molecule and all-atom simulation studies have also demonstrated that monovalent cations can alter DNA helical parameters, including twists, in an ion-specific manner, indicating that even simple alkali ions can fine-tune DNA structure beyond nonspecific charge screening [53].

Divalent cations such as Mg(2+) and Ca(2+) exert stronger effects than monovalent cations because they neutralize DNA charge more efficiently and can form more localized interactions with phosphate oxygens and groove sites. Mg(2+) is important in biological environments, where it contributes to nucleic-acid folding, stabilization, and ion-mediated association. Compared with monovalent ions, divalent cations can reduce DNA–DNA repulsion more effectively and may generate bridging or correlation-mediated attractions under certain conditions [54,55]. However, several studies indicate that divalent ions do not always induce bulk DNA condensation efficiently and may even inhibit condensation induced by trivalent polyamines such as spermidine(3+), depending on ionic competition and counterion entropy [42]. Recent work has shown that divalent alkaline-earth cations can mediate sequence-dependent DNA–DNA attraction, suggesting that divalent-ion effects are controlled not only by valency but also by local DNA structure and sequence-dependent ion binding [56].

Trivalent and higher-valency cations, including polyamines such as spermidine(3+) and spermine(4+), and other multivalent counterions, can induce much more dramatic conformational changes. Multivalent cations can overcompensate the negative charge of DNA, promote attractive interactions between duplexes, and drive DNA condensation into compact toroidal, rod-like, or liquid-crystalline assemblies [56,57]. Theoretical and simulation studies indicate that these effects arise from ion–ion correlations, counterion release, bridging interactions, and charge inversion [58,59,60,61,62,63]. Electrophoretic measurements have provided direct experimental evidence that multivalent counterions can overcompensate the negative charge of condensed DNA. Besteman and co-workers demonstrated that the electrophoretic mobility of DNA condensates can reverse sign in the presence of multivalent ions, indicating charge inversion rather than simple charge neutralization [11].

Several theoretical and experimental studies have examined competitive counterion effects in DNA condensation and higher-order conformational transitions [19,22,42,64,65]. Here, it is noted that the competitive effect between counterions with different valencies contradicts the basic framework of Manning–Oosawa theory [27,28]. Li et al. extended counterion condensation theory to a two-cation-species system and introduced the iso-competition point as a quantitative measure of competitive binding between monovalent and multivalent cations [66]. Burak, Ariel, and Andelman developed a theoretical model for the onset of DNA aggregation in the presence of monovalent salt and multivalent polyamines, showing that the threshold concentration of multivalent cations is strongly affected by monovalent salt concentration and by the number of condensed multivalent counterions [20]. Their subsequent analysis emphasized that the distribution of condensed ions around DNA is highly sensitive to short-range interactions, salt conditions, and model-dependent approximations.

This review/perspective article focuses on the interactions between negatively charged DNA as a semi-flexible polyelectrolyte and various cationic species that modulate the cellular environment, highlighting their close association with conformational changes and emphasizing their physicochemical and electrostatic properties. In particular, this paper highlights the competitive effects of coexisting multiple cations on DNA conformational behavior, as reported by recent experimental findings [42,43,64,67,68].

## 3. Experimentally Observed Competitive Effects in Conformational Changes in DNA Under Coexisting Divalent and Trivalent Cations

A single DNA molecule observation in an aqueous solution by adopting fluorescence microscopy have revealed that long DNA undergoes a discrete coil–globule transition from an extended coil state to a compact conformation [69,70]. Using this methodology, the conformational changes in giant DNA molecules longer than several tens kbp have been observed, which behaves as a semi-flexible polymer in aqueous solution in the presence of multiple cations [42].

**Figure 1:**
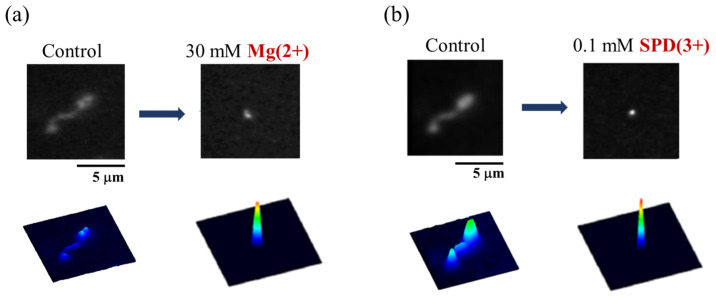
Fluorescence images of T4 GT7 DNA with quasi three-dimensional images of fluorescence intensity. (**a**) Effect of the addition of 30 mM Mg(2+), (**b**) Effect of the addition of 0.1 mM SPD(3+). Adapted from Ref. [42] Tongu, C., et al. *J. Chem. Phys.* 2016; licensed under a Creative Commons Attribution (CC BY) license.

Figure 1 shows fluorescence images of the conformational states of a single T4 GT7 DNA (166 kbp) molecule undergoing translational and intrachain Brownian motion in aqueous solution, as observed by fluorescence microscopy, together with the corresponding quasi-two-dimensional images of fluorescence intensity, revealing the effects of added Mg(2+) and spermidine (SPD(3+)). The experimental results indicate that DNA adopts an elongated conformation in the absence of Mg(2+) and SPD(3+). In contrast, in the presence of 30 mM Mg(2+), DNA exhibits a shrunken structure, which is distinct from the tightly compacted conformations induced by high-valence cations such as SPD(3+). The addition of 0.1 mM SPD(3+) causes a higher-order structural transition from an elongated coil to tightly compact globule states. According to counterion condensation theory, DNA is neutralized up to approximately 75, 85%, and 90% by monovalent, divalent, or trivalent cations in an aqueous solution, respectively, as deduced from the Bjerrum length [27,28,42]. Progressive neutralization of DNA reduces the electrostatic repulsion between DNA segments, thereby promoting its folding transition. Notably, during the folding transition of long semi-flexible DNA, chain stiffness with relatively large persistence length favors the formation of locally parallel alignments between neighboring DNA segments.

**Figure 2:**
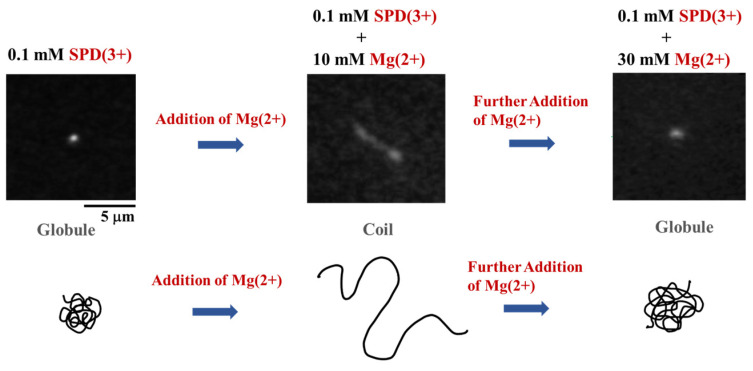
Competitive effects on the conformational change in T4 GT7 DNA in the presence of SPD(3+) and Mg(2+), together with a schematic diagram of DNA conformational change due to the addition of Mg(2+). Adapted from Ref. [42] Tongu, C., et al. *J. Chem. Phys.* 2016; licensed under a Creative Commons Attribution (CC BY) license.

**Figure 3:**
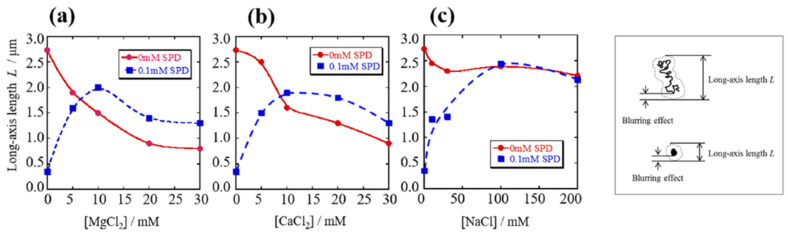
Mean values of the long-axis length L in the absence and presence of SPD(3+), depending on the concentrations of (**a**) MgCl_2_, (**b**) CaCl_2_, and (**c**) NaCl. Adapted from Ref. [42] Tongu, C., et al. *J. Chem. Phys.* 2016; licensed under a Creative Commons Attribution (CC BY) license.

Figure 2 shows fluorescence images of the conformational changes in T4 GT7 DNA in the presence of Mg(2+) and SPD(3+) [42], together with a schematic diagram of DNA conformational changes. The experimental results show that DNA has a compacted globule state in the presence of 0.1 mM SPD(3+), and the addition of 10 mM Mg(2+) induces an unfolding transition from the globule to elongated coil states. Further addition of 30 mM Mg(2+) induces a transition of DNA back to a folded compact conformation. These results indicate that Mg(2+) has an antagonistic effect on DNA compaction induced by SPD(3+). From the viewpoint of DNA–cation electrostatic interactions, this behavior can be understood as follows. DNA has a high negative charge in aqueous solutions because the phosphate groups in its backbone dissociate and release protons, leaving behind negatively charged phosphate ions. Thus, electrostatic interactions between DNA and cations play a decisive role in DNA conformational changes in solutions by neutralizing the negative charge on DNA. Neutralization of DNA induces a conformational transition from an elongated to a folded compacted state by reducing the repulsive electrostatic interactions between DNA segments. Based on the Debye–Hückel theory [71], the electrostatic contributions of cations with different valences are predicted to be always additive through their contribution to the ionic strength, which depends on the ion concentration and the square of the valence. Thus, the addition of Mg(2+) and SPD(3+) is expected to act cooperatively to enhance the degree of DNA charge neutralization, leading to a conformational transition of DNA toward a folded compact state. In contrast, experimental results demonstrate that Mg(2+) and SPD(3+) competitively influence the higher-order structural transitions of DNA [42]. Notably, the observed competitive effect between Mg(2+) and SPD(3+) cannot be explained within the framework of Debye–Hückel theory. Additional experiments have been constructed to examine the competitive effects of coexisting monovalent, divalent, and trivalent cations on DNA conformation. Figure 3 shows mean values of the long-axis length of T4 GT7 DNA in the absence and presence of 0.1 mM SPD(3+) at different concentrations of Mg(2+), Ca(2+) and Na(+), together with a schematic diagram of long-axis length of DNA [42]. DNA compaction occurred at 0.1 mM SPD(3+) when Mg(2+), Ca(2+), and Na(+) were absent from the solution. These results indicate that both Na(+) and Ca(2+) competitively influence DNA conformation in a manner similar to Mg(2+) in the presence of 0.1 mM SPD(3+). These results clearly demonstrate that multiple cations in aqueous solutions competitively influence DNA conformational changes.

**Figure 4:**
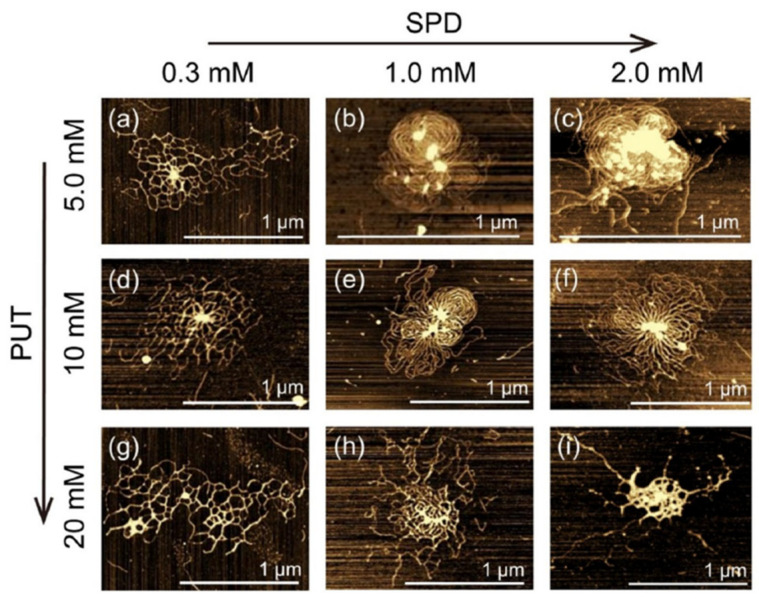
AFM images of T4 GT7 DNA under the coexistence of SPD(3+) and PUT(2+). The SPD(3+) concentrations were fixed at 0.3 mM for (**a**,**d**,**g**), 1.0 mM for (**b**,**c**,**h**), and 2.0 mM for (**c**,**f**,**i**). The PUT(2+) concentrations were fixed at 5.0 mM for (**a**–**c**), 10 mM for (**d**–**f**), and 20 mM for (**g**–**i**). The DNA concentration was fixed at 0.6 μM in the nucleotide units. Reproduced from Ref. [43] Ogawa, H., et al. *Eur. Biophys. J.* 2026; licensed under a Creative Commons Attribution (CC BY) license.

Recently, the mutual effects of diamine putrescine (PUT(2+)) and triamine spermidine (SPD(3+)) on DNA conformational changes have been reported [43]. Polyamines are essential cationic biomolecules with linear molecular structures. Figure 4 shows the differences in the conformation of T4 GT7 DNA at various concentrations of PUT(2+) and SPD(3+) as observed by atomic force microscopy (AFM). A cross-linking mesh-like network was observed in the presence of PUT(2+) and SPD(3+), as shown in Figure 4a,d,g. In contrast, flower-like structures appeared with increasing SPD(3+) concentration in the presence of PUT(2+) (Figure 4b,c,e,f), which caused a loose shrinkage of the DNA conformation with parallel-aligned segments. The experimental results indicate that polyamines with different valences induce distinct higher-order structural changes in DNA.

Experimental findings during the last decade indicated that the coexistence effects of multiple cations, including metal cations and polyvalent polymers, on DNA conformational transitions are highly complex, synergistic, and competitive depending on their concentrations.

## 4. Theoretical Treatment of the Observed Competitive Effects of Multiple Cations with Different Valencies on DNA Conformational Transitions

Electrostatic interactions between DNA and cations play a crucial role in the conformational properties of DNA in aqueous solutions. The regularly spaced negative charges along the DNA backbone interact electrostatically with neighboring charges. Thus, consideration of the specific correlations between negative charges along DNA is essential for understanding DNA–cation interactions. However, such correlations are underestimated in the Debye–Hückel theory, which is based on the mean-field approximation of the electric field by neglecting the correlation effect on long-range Coulombic interaction. Moreover, it is noted that accounting for the translational entropy of the counterion is essential for describing DNA neutralization under conditions involving multiple coexisting cations.

A theoretical framework by adapting the essentials in counterion condensation theory have been reported, which incorporates ion-exchange mechanisms between counterions and multivalent cations in DNA–cation interactions [25,42,72]. In the proposed model, coexisting monovalent cations are treated as counterions of the DNA added to aqueous solution as in the actual experimental procedure, and full charge neutralization is assumed on the tightly compact DNA state for simplicity. The free energy of DNA can be expressed as the sum of several contributions, including the elastic deformation energy, *F_ela_*, the translational entropy of counterions, *F_trans_*, the electrostatic interaction between DNA segments, *F_elec_*, and the intermolecular mixing contribution of DNA molecules, *F_mix_*, as follows [25]:

(1) $$F=F_{ela}+F_{trans}+F_{elec}+F_{mix}$$

With regard to electrostatic interaction, the Debye length is typically approximately ~1 nm under conventional buffered conditions for DNA solutions. This length scale is considerably shorter than the average distance between DNA segments (contour length ~50 nm) in an elongated DNA chain, which is generally greater than 10 nm. In addition, in the globular state, the negative charge of a DNA molecule is largely neutralized by surrounding cations. These considerations suggest that *F_elec_* makes only a minor contribution to the total free energy of DNA under the present theoretical framework, where the translational entropy is evaluated as a separate contribution from Coulombic interactions. The mixing effect with water molecules of the semi-flexible polyelectrolyte chain of DNA, *F_mix_*, is expected to have similar effects as in *F_ela_*. Thus, for simplicity, hereafter, the effects arising from the elastic energy of the DNA chain and the translational entropy of the counterions have been discussed by neglecting the contributions of *F_elec_* and *F_mix_*. In the model, the persistence length of DNA was assumed to remain constant during the conformational transition between the coil and globule states for simplicity. By introducing the total number of phosphate groups $\Gamma_{0}$, the counterion concentration of monovalent cation *C*_1,_ and the degree of counterion condensation *p*, the free energy cost of counterion condensation can be expressed as $p\Gamma_{0}C_{1}$ in units of thermal energy *k*_B_*T*. In the presence of monovalent and trivalent cations, the free energy change arising from the contribution of translational entropy associated with the folding transition is described by the following equation, in which complete DNA charge neutralization is achieved by $\Gamma_{0}/3$ trivalent cations, accompanied by the release of $p\Gamma_{0}$ monovalent cations.

(2) $$\Delta F_{1,\;3}=-\frac{1}{3}\Gamma_{0}\ln C_{3}+p\Gamma_{0}C_{1}\;,\;$$

where *C*_3_ is the trivalent concentration. The ion exchange process between monovalent and trivalent cations reduces the free energy of DNA, reflecting a balance between the translational entropy gain from the release of monovalent cations and the penalty associated with the binding of trivalent cations to DNA. In the coexistence of monovalent, divalent, and trivalent cations, the free energy change in a DNA chain is expressed by the following equation for the fully charge neutralized state with the relationship of $\Gamma_{0}=3\Gamma_{3}+2\Gamma_{2}$.

(3) $$\Delta F_{1,\;2,\;3}=-\Gamma_{3}\ln C_{3}-\Gamma_{2}\ln C_{2}+p\Gamma_{0}C_{1}\;,\;$$

where *C*_2_ is the concentration of divalent cations. By considering the relationship between *Γ*_2_ and *Γ*_3_ with a simple approximation of $\Gamma_{3}/\Gamma_{2}=9C_{3}/4C_{2}$ as in the usual Debye–Hückel theory, Equation (3) is revised as follows:

(4) $$\Delta F_{1,\;2,\;3}=-\frac{4C_{2}}{27C_{3}+8C_{2}}\left(\frac{9C_{3}}{4C_{2}}\ln C_{3}+\ln C_{2}\right)\Gamma_{0}+p\Gamma_{0}C_{1}.\;$$

Based on the Boltzmann distribution for a two-state DNA conformation, that is, the coil and globule states, the probability ratio of these states is given by

(5) $$\frac{p_{g}}{p_{c}}=\exp\left(-\frac{\Delta F}{k_{B}T}\right),\;$$

where ΔF is the difference in free energy between the two states. The probabilities of occupying the coil and globule states, *p*_c_ and *p*_g_, are expressed as follows:

(6) $$p_{c}=\frac{1}{1+\exp\left(-\frac{\Delta F}{k_{B}T}\right)}\;\;,\;\;p_{g}=1-p_{c}\;.\;$$

Based on these arguments, the ensemble average of the long-axis length of DNA molecules, $\bar{L}$, is obtained as follows:

(7) $$\bar{L}=p_{c}L_{c}+p_{g}L_{g},\;$$

where *L*_c_ and *L*_g_ are the long-axis lengths of the DNA in the coil and globule states, respectively.

**Figure 5:**
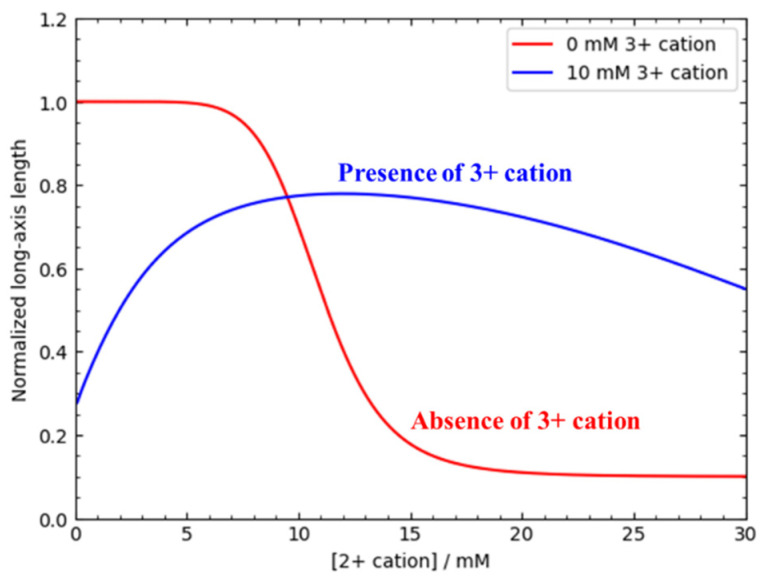
Calculated normalized long-axis length (ensemble average) of DNA molecules based on our model in the absence and presence of trivalent cations, as a function of the concentration of divalent cations. Adapted from Ref. [42] Tongu, C., et al. *J. Chem. Phys.* 2016; licensed under a Creative Commons Attribution (CC BY) license.

**Figure 6:**
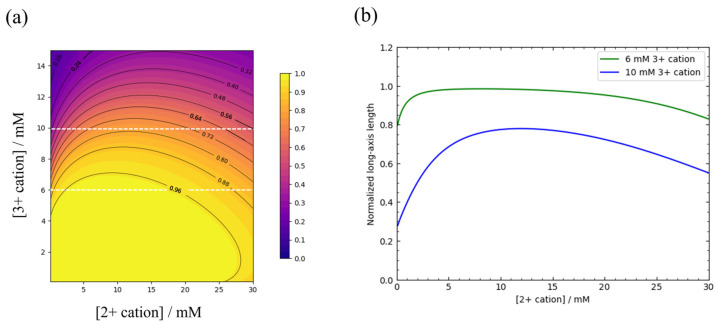
Calculated normalized long-axis length of DNA molecules as a function of divalent and trivalent cation concentrations. This figure was depicted through our calculations based on the theoretical framework given in Section 4 of the present manuscript. (**a**) Contour maps showing the dependence on divalent and trivalent cation concentrations. The results for trivalent cation concentrations of 6 mM and 10 mM are indicated by white dashed lines. (**b**) Normalized long-axis length plotted as a function of divalent cation concentrations in the presence of trivalent cations.

Figure 5 shows the average long-axis length of DNA as a function of divalent cation concentration in the absence and presence of 0.1 mM trivalent cations, using Equation (7) at 300 K. The calculated values are obtained using $\bar{L}/L_{c}$, which are assumed to be 3.0 and 0.3 μm for *L*_c_ and *L*_g_, respectively. The results indicate that the normalized DNA length decreases with increasing divalent cation concentrations in the absence of trivalent cations. In contrast, DNA initially adopts a globular conformation owing to the presence of trivalent cations and is elongated upon the addition of divalent cations. Further increase in the divalent concentration promotes a conformational retransition from the coil to the globule state, showing a trend essentially the same as in experimental results [42]. To demonstrate the characteristic contributions of divalent and trivalent cations to DNA conformational changes as theoretically described in Section 4, the normalized long-axis length of DNA as a function of the concentrations of both divalent and trivalent cations has been calculated, as shown in Figure 6. These results indicate that trivalent cations induce DNA compaction more effectively than divalent cations. Based on the calculated results, the reduction in DNA free energy by trivalent cations is dominant at low concentrations of divalent cations, leading to the folding transition of DNA. Considering the ion exchange process between monovalent counterions and divalent or trivalent cations, two or three monovalent counterions are replaced by divalent or trivalent cations and released from the DNA chain. This implies that the gain in translational entropy associated with ion exchange between counterions and trivalent cations is greater than that for divalent cations. Thus, the addition of divalent cations reduces the translational entropy gain provided by trivalent cations, thereby increasing the free energy of DNA and favoring a conformational transition from the globule to the coiled state. With increasing concentrations of divalent cations, the gain in translational entropy owing to divalent cations becomes progressively dominant, resulting in DNA shrinkage.

Based on these considerations, in the presence of multiple cations, the proposed framework can be extended to higher-valent cations such as tetravalent and pentavalent species, as well as to additional cation types, by incorporating factors such as $\Gamma_{0}/4$, $\Gamma_{0}/5$, and higher-order terms into Equation (3).

## 5. Conclusions

In this review/perspective article, DNA conformational changes in the presence of multiple cations with different valencies have been discussed based on recently reported experimental findings and theoretical analyses. In particular, this review highlights the characteristic effects of coexisting multiple cations, which give rise to antagonistic behavior: the compact DNA conformation induced by the trivalent cation spermidine (SPD(3+)) undergoes a transition to an elongated state upon the addition of the divalent cation Mg(2+). Such antagonistic behavior cannot be explained solely by electrostatic interactions between DNA and cations based on Debye–Hückel-type modelling. This article emphasizes the importance of counterion translational entropy in determining the free energy of DNA and extends the theoretical framework to account the antagonistic effects in DNA conformational changes by incorporating the translational entropy contribution associated with ion exchange between monovalent and multivalent counterions.

In the cellular environment, multiple cationic species, including metal ions and biopolymers, play essential roles in biological functions. Consequently, DNA–cation interactions within cells are expected to be more complex and highly sensitive to variations in the concentrations of multiple cations. Currently reported experimental findings, together with theoretical treatments, provide fundamental insights into DNA conformational transitions under conditions where multiple cations coexist, as commonly observed in living cells. In the above discussion, to focus on the mechanism of characteristic effects among counterions with different valencies, the free energy contributions arising from mixing interactions with water molecules and electrostatic interactions were omitted for simplicity. Incorporating these contributions in future extensions of the model would enable a more comprehensive and quantitative description of the system. Furthermore, the cytoplasmic environment in living cells is highly crowded, comprising approximately 40 wt% of biopolymers, proteins, and other species. The contribution of such crowding [73,74,75] must be considered to obtain a more comprehensive understanding of DNA conformational dynamics within cells.

## Data Availability

This review article includes a small amount of newly generated data. All relevant data supporting the findings are provided in the main text. Further details are available from the corresponding author upon reasonable request.

## Abbreviations

The following abbreviations are used in this manuscript:

AFM	Atomic Force Microscopy
bp	Base Pair(s)
DNA	Deoxyribonucleic Acid
